# Utility of serum immunoglobulin A antibody against glycopeptidolipid core antigen in the diagnosis and management of hypersensitivity pneumonitis associated with *Mycobacterium avium* complex: A case report

**DOI:** 10.1016/j.rmcr.2022.101790

**Published:** 2022-12-09

**Authors:** Yuta Mori, Harunori Nakashima, Takashi Funasaka, Sho Hori, Michiko Kagajo, Takashi Abe, Morihide Ando, Joe Shindoh

**Affiliations:** a Department of Respiratory Medicine, Ogaki Municipal Hospital, Ogaki, Japan; b Department of Respiratory Medicine, Nagoya University Graduate School of Medicine, Nagoya, Japan

**Keywords:** Hypersensitivity pneumonitis, *Mycobacterium avium* complex, Hot-tub lung, Enzyme-linked immunosorbent assay, BAL, bronchoalveolar lavage, CRP, C-reactive protein, DL_CO_, diffusing capacity of the lung for carbon monoxide, ELISA, enzyme-linked immunosorbent assay, FVC, forced vital capacity, GPL, glycopeptidolipid, HP, hypersensitivity pneumonitis, HRCT, high-resolution computed tomography, MAC, *Mycobacterium avium* complex, PaO_2_, partial pressure of oxygen, WBC, white blood cell

## Abstract

Measurement of the levels of serum immunoglobulin A antibody against glycopeptidolipid (GPL) core antigen, a cell surface antigen found in *Mycobacterium avium* complex (MAC), has been reported to be useful in the diagnosis and management of pulmonary MAC infection. However, evidence on its utility in hypersensitivity pneumonitis (HP) associated with MAC (i.e., “hot-tub lung”) is limited. We herein report a case of HP associated with MAC in which the GPL core antibody levels were serially measured from diagnosis to treatment and thereafter. A 61-year-old man was suspected to have non-fibrotic HP based on the clinical course, laboratory findings, imaging pattern, bronchoalveolar lavage (BAL) lymphocytosis, and histopathological findings. Based on the history of whirlpool bath use, inhalation of aerosolized MAC was suspected as the cause of HP. The GPL core antibody level, measured using an enzyme-linked immunosorbent assay kit, was elevated, suggesting an immunological sensitization to MAC. A provocation test using the patient's whirlpool bath was positive. An identical MAC strain was isolated from the BAL fluid and bathtub. Accordingly, the patient was diagnosed with HP caused by the inhalation of aerosolized MAC from the whirlpool bath. The patient recovered after steroid treatment and discontinuation of the whirlpool bath. The GPL core antibody levels decreased with disease improvement. In conclusion, GPL core antibody levels could be elevated in HP associated with MAC and decrease with disease improvement. Thus, measurement of the GPL core antibody level may be useful for the diagnosis and management of HP associated with MAC.

## 1 Introduction

Hypersensitivity pneumonitis (HP) is an immune-mediated interstitial lung disease caused by repeated inhalation of antigens [1]. Accurate antigen identification is fundamental for the diagnosis and management of HP; however, it is often difficult to achieve. To identify the causative antigen, it is important to perform a comprehensive evaluation, including a detailed history of exposure, environmental assessment, response to antigen avoidance, serum antigen-specific antibody testing, and antigen-specific inhalation challenge testing [1]. The presence of serum-specific antibodies against a specific antigen is indicative of previous exposure and immunologic sensitization to that antigen and may increase the probability of HP associated with that antigen [2]. The efficacy of serum antigen-specific antibody testing in the diagnosis of HP has been reported, especially for specific antibodies against avian antigens [3].

Inhalation of aerosolized *Mycobacterium avium* complex (MAC) from water sources can cause HP, more commonly known as “hot-tub lung” based on its typical source [4]. Although the causative antigens of HP vary by geographic region, a previous report in the USA showed that MAC was the second most common cause of HP after avian antigens, accounting for 28% of patients with the identified antigen [5]. Nevertheless, few studies have examined the efficacy of serum antigen-specific antibody testing in HP associated with MAC. In pulmonary MAC infection, measurement of the levels of serum immunoglobulin A antibody against glycopeptidolipid (GPL) core antigen, a cell surface antigen found in MAC, has been reported to be useful for diagnosis and management [[6], [7], [8], [9], [10]], and its efficacy is mentioned in the British Thoracic Society guidelines for the management of non-tuberculous mycobacterial pulmonary disease [11]. However, little is known regarding its utility in HP associated with MAC. We herein describe the case of a patient in whom inhalation of aerosolized MAC from a whirlpool bath at home caused HP, and the GPL core antibody levels were serially measured from diagnosis to treatment and thereafter.

## 2 Case presentation

A 61-year-old man was referred to our hospital in May 2021 because of progressive dyspnea on exertion and productive cough of 2 months’ duration. He was a current smoker (1 pack per day for 41 years) and had no significant medical history. He had retired from the electronics industry in March 2020. He spent most of his time at home after retirement. He completely remodeled his 35-year-old reinforced-concrete house in October 2020. His house was well-cleaned and free of mold. He did not own pets.

On arrival, his body temperature was 36.4 °C; blood pressure, 120/70 mmHg; pulse rate, 95 bpm; and oxygen saturation, 92% on room air. Physical examination revealed inspiratory crackles in both lungs. Arterial blood gas analysis in room air showed a partial pressure of carbon dioxide of 39.4 mmHg and partial pressure of oxygen (PaO_2_) of 71.7 mmHg. The white blood cell (WBC) count was within the reference range. The serum levels of C-reactive protein (CRP) (1.61 mg/dL), Krebs von den Lungen-6 (2373 U/mL), and surfactant protein-D (517.9 ng/mL) were elevated.

**Fig. 1:**
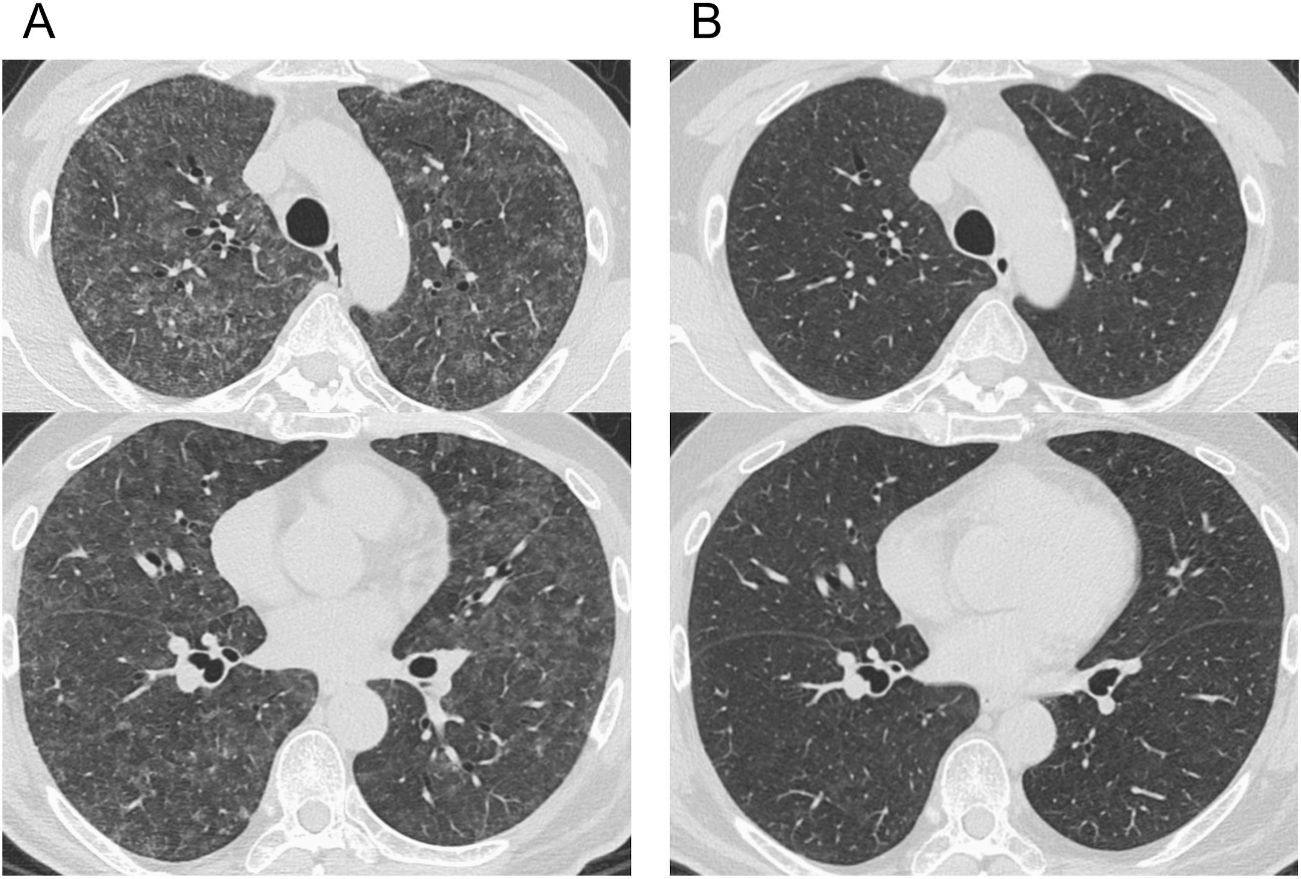
High-resolution computed tomography (HRCT) images of the lung. (A) HRCT on admission showing diffuse ground-glass opacities with ill-defined centrilobular nodules throughout both lungs with upper lobe predominance. (B) Complete resolution of the abnormalities after 3 months of steroid treatment and discontinuation of the whirlpool bath.

High-resolution computed tomography (HRCT) of the chest showed diffuse ground-glass opacities with ill-defined centrilobular nodules throughout both lungs with upper lobe predominance, without cavitation, bronchiectasis, or tree-in-bud opacities (Fig. 1A). Pulmonary function tests showed a restrictive pattern with a decreased predicted value for forced vital capacity (FVC) and decreased predicted value for diffusing capacity of the lung for carbon monoxide (DL_CO_) (FVC % predicted, 68.5%; DL_CO_ % predicted, 62.6%).

**Fig. 2:**
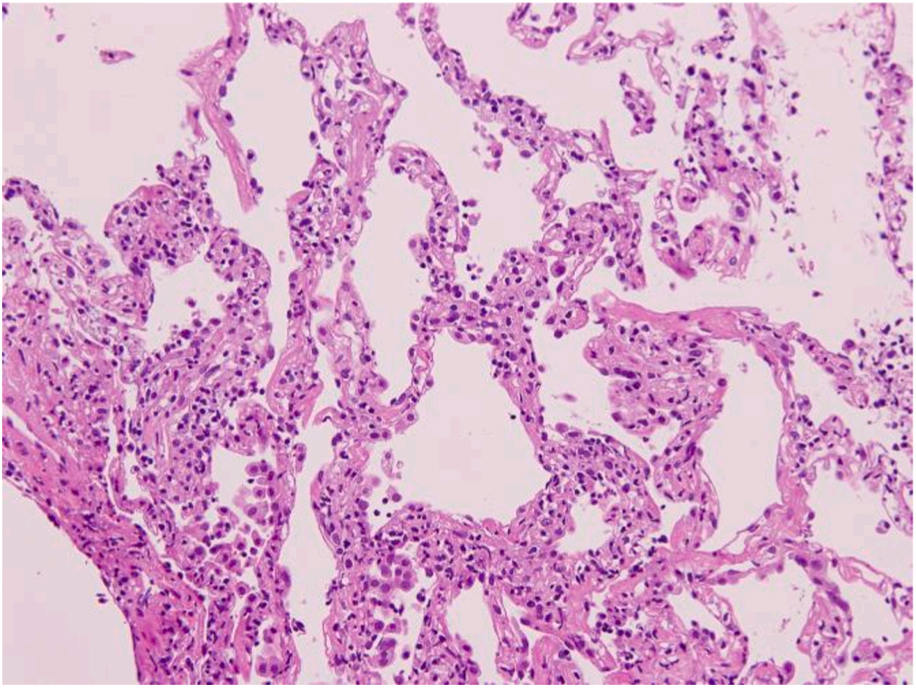
Transbronchial lung biopsy showing alveolitis with infiltration of lymphocytes without granulomas (hematoxylin and eosin, original magnification × 200).

Based on the clinical course and examination findings, the patient was suspected to have non-fibrotic HP associated with potential antigens at home. The patient was admitted for further evaluation and to avoid possible causative antigens present at home. A flexible bronchoscopy was performed. The bronchoalveolar lavage (BAL) fluid showed an elevated total cell count (4.3 × 10^5^/mL) with an increased percentage of lymphocytes (75.0%), and the ratio of CD4^+^ to CD8^+^ cells was increased to 5.58. Histopathological examination of the transbronchial lung biopsy specimen revealed alveolitis with infiltration of lymphocytes without granulomas (Fig. 2). These results further increased the probability of non-fibrotic HP.

A careful environmental history revealed that the patient had added a whirlpool function to the bathtub when he remodeled his home in October 2020 and had been using it frequently since that winter. Based on his exposure history, aerosols from the whirlpool bath at home, more specifically aerosols containing MAC, were suspected to be the cause of HP. To identify immunologic sensitization to MAC, GPL core antibody levels were measured using an enzyme-linked immunosorbent assay (ELISA) kit (TAUNS Laboratories, Shizuoka, Japan). The antibody level was elevated to 4.22 U/mL and was positive according to a diagnostic cut-off value of 0.7 U/mL for pulmonary MAC infection. The positive result for the GPL core antibody was considered to increase the probability that MAC was the causative antigen; hence, aerosols from the whirlpool bath were the cause of HP.

The patient's clinical symptoms gradually improved without treatment within a week of admission. After 2 weeks of hospitalization, his CRP level decreased, and FVC and DL_CO_ improved; however, PaO_2_ and radiographic findings did not improve. To determine whether aerosols from the whirlpool bath at home caused HP, we conducted two returning-home provocation tests: one with and one without the use of the whirlpool bath when bathing (showering and simply soaking in the bathtub were performed in both tests). In the returning-home provocation test without the use of the whirlpool bath, no significant changes were observed in his symptoms or examination findings. In contrast, in the returning-home provocation test with the use of the whirlpool bath, the patient developed dyspnea and productive cough 6 h after the use of the whirlpool bath. The following day, the patient had a slight fever, decreased PaO_2_, increased WBC counts and CRP levels, decreased FVC and DL_CO_, and increased radiographic abnormalities. The returning-home provocation test using the whirlpool bath was considered positive.

The culture from the BAL fluid ultimately resulted in the growth of *M. avium* after 20 days of incubation. Upon surveying the patient's home, *M. avium* was isolated from the drain and the whirlpool jets of the bathtub. The variable number of tandem repeat typing analyses revealed that the *M. avium* strains isolated from the BAL fluid and bathtub were identical. Accordingly, the patient was diagnosed with HP caused by the inhalation of aerosolized MAC from the whirlpool bath at home.

The patient was advised to discontinue use of the whirlpool bath and was treated with steroids (0.5 mg/kg/day of oral prednisolone) with no antimicrobial therapy. The patient recovered rapidly and was discharged after 26 days of hospitalization. Thereafter, the steroids were tapered over the next 3 months. His symptoms completely resolved, and his laboratory and pulmonary function test results were almost normalized over this period. Repeat chest HRCT after steroid treatment demonstrated complete resolution of the abnormalities (Fig. 1B). After discontinuing the use of the whirlpool bath, the clinical symptoms and examination findings continued to improve even after the completion of steroid treatment.

The patient's clinical course is presented in Table 1. Despite antigen avoidance during the 2 weeks of hospitalization, the GPL core antibody levels increased from the time of admission. The antibody levels did not vary significantly before and after each of the two returning-home provocation tests. The antibody levels decreased after steroid treatment and discontinuation of the whirlpool bath and remained low even after the completion of steroid treatment.

**Table 1:** Changes in body temperature, laboratory data, and pulmonary function test results.

Variable	Value					
	On admission	After 2 weeks of hospitalization (pre-provocation test)	Post-provocation test without the use of the whirlpool bath	Post-provocation test with the use of the whirlpool bath	After 3 months (at the end of steroid treatment)	After 6 months
Body temperature, °C	36.4	36.5	36.2	37.0	36.4	36.2
PaO_2_, mmHg	71.7	63.0	72.0	64.5	NA	NA
WBC, /mm^3^	9230	6520	7390	19,510	8520	10,060
CRP, mg/dL	1.61	0.12	0.12	3.20	0.26	0.42
FVC, % predicted	68.5	79.1	75.5	53.9	77.8	79.8
DL_CO_, % predicted	62.6	68.0	NA	58.8	77.6	NA
GPL core antibody, U/mL	4.22	8.20	8.49	8.97	1.81	1.93

CRP, C-reactive protein; DL_CO,_ diffusing capacity of the lung for carbon monoxide; FVC, forced vital capacity; GPL, glycopeptidolipid; NA, not available; PaO_2_, partial pressure of oxygen; WBC, white blood cell.

## 3 Discussion

To the best of our knowledge, this is the first case report in which GPL core antibody levels were serially measured from diagnosis to treatment and thereafter in a patient with HP associated with MAC. Importantly, we demonstrated two crucial points: GPL core antibody levels may be elevated in HP associated with MAC and decrease with disease improvement.

First, we found that the GPL core antibody level can be elevated in HP associated with MAC. GPL is a highly antigenic lipid on the surface of the MAC cell wall and is absent in the other major *Mycobacterium* species, such as *M. tuberculosis* or *M. kansasii* [12]. The ELISA kit for measuring the GPL core antibody levels was developed in Japan and is currently used as a diagnostic aid for pulmonary MAC infection. Several studies evaluating the usefulness of the ELISA kit for the diagnosis of pulmonary MAC infection have reported its high specificity, with sensitivity ranging from 52% to 85% and specificity from 91% to 100% at a cut-off value of 0.7 U/mL [[6], [7], [8], [9]]. In addition, a multicenter study in Japan showed that the GPL core antibody levels were not elevated in patients whose MAC had merely colonized the respiratory tract [6]. These results suggest that patients with elevated GPL core antibody levels are more likely to have MAC-associated lung diseases. Although most previous reports have validated the GPL core antibody in patients with pulmonary MAC infections, our patient showed that the GPL core antibody level can be elevated even in HP associated with MAC. We are convinced that the diagnosis in our patient was non-fibrotic HP and that the causative antigen was MAC. Based on the diagnostic criteria of the American Thoracic Society, Japanese Respiratory Society, and Asociación Latinoamericana del Tórax clinical practice guideline on the diagnosis of HP in adults [1], the combination of exposure assessment, HRCT findings, BAL lymphocytosis, and histopathological findings in our patient provided evidence for the definite diagnosis of non-fibrotic HP. In addition, we concluded that MAC was the causative antigen because the provocation test with the use of the whirlpool bath was considered positive, an identical strain of MAC was detected in the BAL fluid and bathtub, and no recurrence was observed after discontinuing the use of the whirlpool bath.

One previous report has described the GPL core antibody levels in patients with hot-tub lung [13]. In that report, a married couple was simultaneously affected by hot-tub lung: the husband had elevated GPL core antibody levels, whereas the wife did not. The result observed in the husband supports our finding that the GPL core antibody level can be elevated in HP associated with MAC. However, there is one difference from our patient: the HRCT findings of the husband showed nodular shadows and bronchiectasis, suggestive of the coexistence of MAC infection. Although HP associated with MAC is understood as a condition in which MAC infection and hypersensitivity features can coexist [4], in our patient, the component of infectious lung disease seemed to be infinitesimal. This is because the HRCT findings of our patient on admission did not show findings suggestive of MAC infection, such as cavitation, bronchiectasis, or tree-in-bud opacities, and the abnormalities completely resolved after antigen avoidance and steroid treatment without antimicrobial therapy. Moreover, although patients with hot-tub lung often present histologically with well-formed granulomas, which are atypical for classic HP and suggestive of concomitant inflammation due to the infectious process [14], the histopathologic findings in our patient did not show granulomas. To the best of our knowledge, our patient is the first in whom it was shown that the GPL core antibody level was elevated even in inflammation caused by HP alone without an infectious process. In contrast, the wife in the previous report [13] did not show increased GPL core antibody levels. The following possibilities were considered. (1) Other antigens may have caused HP. MAC was not isolated from the respiratory specimen of the wife. Although rare, there have been reports of hot-tub lung caused by antigens other than MAC [15]. (2) The ELISA kit measuring the GPL core antibody may lack sensitivity for the diagnosis of HP associated with MAC, as the sensitivity of this ELISA kit has been reported to be somewhat low in studies of patients with pulmonary MAC infection [[6], [7], [8], [9]]. Further studies should be conducted to evaluate the diagnostic accuracy of GPL core antibodies in HP associated with MAC.

The presence of GPL core antibodies in patients with HP may increase the probability of MAC being the causative antigen. Therefore, measurement of GPL core antibody levels may help identify the causative antigen of HP. Although HP caused by inhalation of aerosolized MAC is better known as hot-tub lung, it can also occur in areas where hot tubs are not widely available. MAC is a ubiquitous environmental organism, mostly found in water and soil [11]. In living environments, MAC has been found in water systems, such as the water supply, plumbing, bathtubs, and showerheads [16]. MAC can survive, persist, and grow in water systems because it is relatively resistant to high temperatures, adheres to solid substrates in aquatic environments because of its cell surface hydrophobicity, and is highly resistant to disinfectants used in water treatment such as chlorine and ozone [17]. Additionally, MAC is readily aerosolized from water to air owing to its cell surface hydrophobicity [17]. Aerosolization results in the production of particles that contain a high density of MAC that are sufficiently small to enter the human alveoli [17]. Therefore, the combination of a water system inhabited by MAC and the aerosol generation mechanism can cause HP. Although most reports of HP associated with MAC are related to exposure from hot tubs, cases related to exposure from spas, showers, and indoor swimming pools have also been reported [18]. In our patient, the cause was exposure from a whirlpool bath at the patient's home. In addition, an outbreak of occupational HP associated with MAC has been reported among hotel technicians engaged in the maintenance of bath facilities [19]. If aerosol-generating water systems other than hot tubs are the cause of HP, it may be difficult to identify the antigen sources via history examination. The presence of GPL core antibodies in a patient with an unidentified antigen may prompt re-evaluation of the patient's living environment for any overlooked aerosol-generating water systems.

Isolation of MAC from respiratory specimens of patients with HP may also increase the probability of MAC being the causative antigen. However, sputum culture was reported to be positive for MAC in only approximately 70% of patients with HP associated with MAC [18]. In addition, although the BAL culture may have a higher isolation rate, bronchoscopy may not be feasible in some situations. Measurement of GPL core antibody levels is highly feasible and may support the diagnosis of HP associated with MAC from a different aspect than isolation of MAC from respiratory specimens.

Second, in our patient, GPL core antibody levels decreased with disease improvement. Previous studies on patients with pulmonary MAC infection have reported that GPL core antibody levels were associated with the extent of the disease as seen on imaging and decreased with treatment such as antimicrobial therapy and surgery [9,10,20]. These results suggest that the GPL core antibody level may be associated with the bacterial load. Therefore, serial measurements of GPL core antibody levels can be used to monitor disease activity and the therapeutic effect on pulmonary MAC infections. Based on the results in our patient, serial measurements of GPL core antibody levels may be an objective indicator of the therapeutic effect even in HP associated with MAC. Moreover, serial measurements of GPL core antibody levels may help objectively assess whether patients continue to appropriately avoid MAC exposure. In contrast, the GPL core antibody levels did not decrease after 2 weeks of antigen avoidance and did not increase after the provocation test. Thus, measurement of GPL core antibody levels may not be useful as an indicator of the response to short-term antigen avoidance or as a monitoring parameter for provocation testing.

## 4 Conclusion

• GPL core antibody levels may be elevated in HP associated with MAC and decrease with disease improvement.
• Thus, measurement of the GPL core antibody level may be useful for the diagnosis and management of HP associated with MAC.
• Further studies should be conducted to evaluate the diagnostic accuracy of GPL core antibodies in HP associated with MAC.

## Funding

This research did not receive any specific grant from funding agencies in the public, commercial, or not-for-profit sectors.

## Author contributions

YM managed the case and drafted the original manuscript. HN, TF, SH, MK, TA, MA, and JS assisted with redaction, correction, and reconstruction of the manuscript. All authors read and approved the final manuscript.

## Patient consent for publication

Informed consent was obtained from the patient.

## Data statement

All relevant data are included in the manuscript**.**

## Declaration of competing interest

None.

## References

[1] Raghu G., Remy-Jardin M., Ryerson C.J., Myers J.L., Kreuter M., Vasakova M., Bargagli E., Chung J.H., Collins B.F., Bendstrup E., Chami H.A., Chua A.T., Corte T.J., Dalphin J.C., Danoff S.K., Diaz-Mendoza J., Duggal A., Egashira R., Ewing T., Gulati M., Inoue Y., Jenkins A.R., Johannson K.A., Johkoh T., Tamae-Kakazu M., Kitaichi M., Knight S.L., Koschel D., Lederer D.J., Mageto Y., Maier L.A., Matiz C., Morell F., Nicholson A.G., Patolia S., Pereira C.A., Renzoni E.A., Salisbury M.L., Selman M., Walsh S.L.F., Wuyts W.A., Wilson K.C. (2020). Diagnosis of hypersensitivity pneumonitis in adults. An official ATS/JRS/ALAT clinical practice guideline. Am. J. Respir. Crit. Care Med..

[2] Johannson K.A., Barnes H., Bellanger A.P., Dalphin J.C., Fernández Pérez E.R., Flaherty K.R., Huang Y.T., Jones K.D., Kawano-Dourado L., Kennedy K., Millerick-May M., Miyazaki Y., Morisset J., Morell F., Raghu G.R., Robbins C., Sack C.S., Salisbury M.L., Selman M., Vasakova M., Walsh S.L.F., Rose C.S. (2020). Exposure assessment tools for hypersensitivity pneumonitis. An official American Thoracic Society workshop report. Ann. Am. Thorac. Soc..

[3] Shirai T., Tanino Y., Nikaido T., Takaku Y., Hashimoto S., Taguchi Y., Baba T., Ogura T., Kataoka K., Nakayama M., Yamada Y., Matsushima S., Nakayama S., Miyazaki Y. (2021). Screening and diagnosis of acute and chronic bird-related hypersensitivity pneumonitis by serum IgG and IgA antibodies to bird antigens with ImmunoCAP. Allergol. Int..

[4] Pennington K.M., Vu A., Challener D., Rivera C.G., Shweta F.N.U., Zeuli J.D., Temesgen Z. (2021). Approach to the diagnosis and treatment of non-tuberculous mycobacterial disease. J. Clin. Tuberc. Other Mycobact. Dis..

[5] Hanak V., Golbin J.M., Ryu J.H. (2007). Causes and presenting features in 85 consecutive patients with hypersensitivity pneumonitis. Mayo Clin. Proc..

[6] Kitada S., Kobayashi K., Ichiyama S., Takakura S., Sakatani M., Suzuki K., Takashima T., Nagai T., Sakurabayashi I., Ito M., Maekura R., MAC Serodiagnosis Study Group (2008). Serodiagnosis of Mycobacterium avium-complex pulmonary disease using an enzyme immunoassay kit. Am. J. Respir. Crit. Care Med..

[7] Kitada S., Levin A., Hiserote M., Harbeck R.J., Czaja C.A., Huitt G., Kasperbauer S.H., Daley C.L. (2013). Serodiagnosis of Mycobacterium avium complex pulmonary disease in the USA. Eur. Respir. J..

[8] Jeong B.H., Kim S.Y., Jeon K., Lee S.Y., Shin S.J., Koh W.J. (2013). Serodiagnosis of Mycobacterium avium complex and Mycobacterium abscessus complex pulmonary disease by use of IgA antibodies to glycopeptidolipid core antigen. J. Clin. Microbiol..

[9] Kitada S., Yoshimura K., Miki K., Miki M., Hashimoto H., Matsui H., Kuroyama M., Ageshio F., Kagawa H., Mori M., Maekura R., Kobayashi K. (2015). Validation of a commercial serodiagnostic kit for diagnosing pulmonary Mycobacterium avium complex disease. Int. J. Tubercul. Lung Dis..

[10] Fukushima K., Kitada S., Matsumoto Y., Komukai S., Kuge T., Kawasaki T., Matsuki T., Motooka D., Tsujino K., Miki M., Miki K., Nakamura S., Kida H. (2021). Serum GPL core antibody levels are associated with disease activity and treatment outcomes in Mycobacterium avium complex lung disease following first line antibiotic treatment. Respir. Med..

[11] Haworth C.S., Banks J., Capstick T., Fisher A.J., Gorsuch T., Laurenson I.F., Leitch A., Loebinger M.R., Milburn H.J., Nightingale M., Ormerod P., Shingadia D., Smith D., Whitehead N., Wilson R., Floto R.A. (2017). British Thoracic Society guidelines for the management of non-tuberculous mycobacterial pulmonary disease (NTM-PD). Thorax.

[12] Chatterjee D., Khoo K.H. (2001). The surface glycopeptidolipids of mycobacteria: structures and biological properties. Cell. Mol. Life Sci..

[13] Kitahara Y., Araki Y., Nakano K. (2016). A case of familial hot tub lung. Respir. Med. Case Rep..

[14] Khoor A., Leslie K.O., Tazelaar H.D., Helmers R.A., Colby T.V. (2001). Diffuse pulmonary disease caused by nontuberculous mycobacteria in immunocompetent people (hot tub lung). Am. J. Clin. Pathol..

[15] Wethasinghe J., Hotu S., Taylor S., Anderson G., Wong C. (2015). Mycobacterium phocaicum and Mycobacterium avium-intracellulare in a patient with hot tub lung. Respirol. Case Rep..

[16] Falkinham J.O. (2013). Ecology of nontuberculous mycobacteria--where do human infections come from?. Semin. Respir. Crit. Care Med..

[17] Falkinham J.O. (2003). Mycobacterial aerosols and respiratory disease. Emerg. Infect. Dis..

[18] Sood A., Sreedhar R., Kulkarni P., Nawoor A.R. (2007). Hypersensitivity pneumonitis-like granulomatous lung disease with nontuberculous mycobacteria from exposure to hot water aerosols. Environ. Health Perspect..

[19] Fjällbrant H., Akerstrom M., Svensson E., Andersson E. (2013). Hot tub lung: an occupational hazard. Eur. Respir. Rev..

[20] Kitada S., Maekura R., Toyoshima N., Naka T., Fujiwara N., Kobayashi M., Yano I., Ito M., Kobayashi K. (2005). Use of glycopeptidolipid core antigen for serodiagnosis of mycobacterium avium complex pulmonary disease in immunocompetent patients. Clin. Diagn. Lab. Immunol..

